# Guidelines for uveal melanoma: a critical appraisal of systematically identified guidelines using the AGREE II and AGREE-REX instrument

**DOI:** 10.1007/s00432-020-03141-w

**Published:** 2020-02-08

**Authors:** Theresa Steeb, Kinan M. Hayani, Paul Förster, Raffael Liegl, Frédéric Toussaint, Max Schlaak, Carola Berking, Markus V. Heppt

**Affiliations:** 1 Department of Dermatology, Universitätsklinikum Erlangen, Friedrich-Alexander-University Erlangen-Nuremberg (FAU), Ulmenweg 18, 91054 Erlangen, Germany; 2 Department of Dermatology and Allergy, University Hospital, Ludwig-Maximilians-University, Frauenlobstr. 9-11, 80337 Munich, Germany; 3 Department of Ophthalmology, University Hospital, Ludwig-Maximilians-University, Mathildenstr. 8, 80336 Munich, Germany

**Keywords:** Uveal melanoma, Orphan disease, Ocular melanoma, AGREE, Guideline, Practice guideline

## Abstract

**Purpose:**

Clinical practice guidelines provide recommendations for the management of diseases. In orphan conditions such as uveal melanoma (UM), guideline developers are challenged to provide practical and useful guidance even in the absence of high-quality evidence. Here, we assessed the methodological quality and identified deficiencies of international guidelines on UM as a base for future guideline development.

**Methods:**

A systematic search was carried out in guideline databases, Medline and Embase until 27th May 2019 for guidelines on UM published between 2004 and 2019. Five independent reviewers assessed the methodological quality of the identified guidelines using the instruments “Appraisal of Guidelines for Research and Evaluation II” (AGREE II) and AGREE-REX (Recommendation EXcellence). Descriptive analysis was performed and subgroup differences were explored with the Kruskal–Wallis (H) test. The relationship between the individual domains and items of the instruments were examined using Spearman’s correlation.

**Results:**

Five guidelines published from 2014 to 2018 by consortia of the United States of America, Canada and the United Kingdom (UK) were included. The highest scores were obtained by the UK guideline fulfilling 48–86% of criteria in AGREE II and 30–60% for AGREE-REX. All guidelines showed deficiencies in the domains “editorial independence”, “applicability”, and “recommendation”. Subgroup differences were identified only for the domain “editorial independence”.

**Conclusion:**

The UK guideline achieved the highest scores with both instruments and may serve as a basis for future guideline development in UM. The domains “editorial independence”, “recommendation”, and “applicability” were identified as methodological weaknesses and require particular attention and improvement in future guidelines.

**Electronic supplementary material:**

The online version of this article (10.1007/s00432-020-03141-w) contains supplementary material, which is available to authorized users.

## Introduction

Uveal melanoma (UM) represents one of the most common ocular malignancies and accounts for about 5% of all melanomas. Primary tumors originate from the pigment cells of the choroid layer, the ciliary body or iris of the eye (Chattopadhyay et al. 2016). With an incidence of 4–7 cases per million in Europe, it is much rarer than cutaneous melanoma (Mallone et al. 2012). Typical driver mutations of cutaneous melanoma in the BRAF and NRAS genes are not found in UM. Instead, more than 80% harbour mutations in the guanine nucleotide binding protein Q polypeptide (GNAQ) and alpha-11 (GNA11) genes (Onken et al. 2008; Van Raamsdonk et al. 2009, 2010), leading to constitutive activation of the MAPK signalling pathway (Shoushtari and Carvajal 2014). Primary disease can be effectively controlled by several local therapy options, however, more than 50% of all UM patients develop distant metastases, predominantly to liver and lungs (Bedikian 2006). Therefore, several liver-directed treatment approaches have been developed, but failed to demonstrate an overall survival benefit (Agarwala et al. 2014). Once UM becomes metastatic, therapy options are limited and have been adopted mostly from cutaneous melanoma despite its clinical and genetic heterogeneity (Heppt et al. 2017b; Steeb et al. 2018).

Clinicians usually rely on evidence-based clinical guidelines for decision-making. Clinical practice guidelines include statements and recommendations intended to optimise patient care, which are informed by a systematic review of evidence and an assessment of the benefits and harms of alternative care options (Graham et al. 2011). Several organizations from dermatological, oncological and ophthalmological societies have published guidelines for the treatment of UM (Nathan et al. 2015; Simpson et al. 2014; Weis et al. 2016). The management of UM is subject to country-specific health care conditions, which must be taken into consideration. As more and more guidelines on UM are being published, users often face multiple guidelines on the same topic, available from different consortia. Numerous methodologies have been developed for the assessment of guidelines (Brouwers et al. 2019; Rico Iturrioz et al. 2004; Zeng et al. 2015). Among these, the most widely applied and validated assessment tool is the “Appraisal of Guidelines for Research and Evaluation (AGREE) II”, which was also favoured by WHO (Dans and Dans 2010; Vlayen et al. 2005). AGREE II was published in 2009 as a revised version of the original AGREE instrument issued in 2001. It comprises 23 items grouped into 6 domains and 2 overall assessment items (Dans and Dans 2010). Recently, “AGREE-REX: Recommendation Excellence” has been launched as a complement to the AGREE II (AGREE-REX Research Team 2019). AGREE-REX is a newly developed tool for the evaluation of the clinical credibility and implementability of practice guidelines and a strategy to inform their development and reporting. It consists of 9 items grouped into 3 domains as well as 2 overall assessment items.

In this article, we critically appraise UM guidelines, which were identified in a systematic literature search and determine their methodological quality using the instruments AGREE II and AGREE-REX. Identifying possible weaknesses and strengths may help to improve future guideline work for this orphan disease and set a framework for an improved future treatment guideline on UM.

## Methods

### Eligibility criteria

Published national and international guidelines on UM were eligible for our appraisal. To provide an appraisal of the most recent and up-to-date guidelines, we only included those that have been published within the previous 5 years. Besides, guidelines had to be published in English or German language. As UM is an orphan disease, we included all guidelines irrespective of their methodological level and their development process. Hence, we also included informal expert statements that are neither based on a systematic assessment of the literature nor on a structured consensus process.

### Search strategy and guideline selection

A systematic search for guidelines was carried out in guideline databases, including multidisciplinary guideline providers and subject-specific guideline providers (Supplementary Table S1). The key search terms included “uveal melanoma”, “ocular melanoma”, “iris melanoma”, “choroidal melanoma”, “ciliary body melanoma” and the German translation “Aderhautmelanom”. Additionally, Medline and Embase (both via Ovid) were searched until 27 May 2019. The detailed search strategy is presented in Supplementary Table S2. After the elimination of double hits, two authors (MVH, TS) independently screened the titles and abstracts of the records that were identified in the databases for eligibility. For records that were considered potentially relevant, the full-text guidelines were obtained, and the inclusion and exclusion criteria were applied. Whenever discrepancies arose, resolution was achieved by discussion with a third independent author (CB).

### Data extraction and rating of the guidelines

Information on each included guideline regarding title, national authority/author, country of origin, publication date, methodological approach and scope were collected and summarized by two authors independently (TS, MVH).

AGREE II was used by five independent reviewers to assess the methodological quality of each guideline identified in the search on a 7-point scale ranging from 1 (strongly disagree) to 7 (strongly agree). Six domains with 23 items were assessed, including: scope and purpose (domain 1), stakeholder involvement (domain 2), rigor of development (domain 3), clarity of presentation (domain 4), applicability (domain 5), and editorial independence (domain 6). Based on the scores of the 6 domains, overall assessment was obtained to assess the quality of the guidelines. The different domains were followed by a general judgement of the guideline’s overall quality considering the evaluated criteria on a 7-point scale from “lowest possible quality” to “highest possible quality”. Furthermore, the evaluator was asked for an answer on the statement “I would recommend this guideline for use” (“yes”, “yes, with modifications” and “no”). The evaluations were performed independently and blinded towards the other evaluators’ assessments using the platform provided by AGREE (my agree plus on https://www.agreetrust.org/).

As a complement to AGREE II, the instrument AGREE-REX was used for the evaluation of the domains clinical credibility and implementability of the guidelines. AGREE-REX includes the 3 domains clinical applicability (domain 1), values and preferences (domain 2) and implementability (domain 3) with 9 items that must be considered to ensure that guideline recommendations are of high quality. This instrument was used by the same five independent authors who rated the quality of the credibility and implementability on a 7-point scale ranging from 1 (lowest quality) to 7 (highest quality). Furthermore, the evaluator was asked for an answer on the recommendation of this guideline in the appropriate context or in the reviewers’ context. The evaluations were performed independently and blinded towards the other evaluators’ assessments using an internally piloted data extraction spreadsheet using Microsoft Excel 2010.

### Analysis

Domain scores were calculated as suggested by the AGREE II and AGREE-REX instructions as a sum of the scores of all evaluators’ assessments of the individual items in the domain for both instruments. The total scores for each domain were then expressed as a percentage of the maximum possible score for that domain. Hence, the range of possible evaluations was 0–100%, with 0% and 100% representing the worst and best possible rating for each domain, respectively.

Statistical analyses were conducted using SPSS (IBM SPSS Statistics version 24, IBM Corporation, Armonk, NY, USA). Descriptive analyses included mean (± standard deviation, SD) or median and interquartile ranges (IQR). Subgroup differences were explored with the Kruskal–Wallis (H) test. The relationship between the individual domains were examined using Spearman’s correlation. A significance level of 0.05 was considered statistically significant. The interrater agreement of the five reviewers was determined using Fleiss’ Kappa (Landis and Koch 1977).

## Results

### Guideline identification

**Fig. 1:**
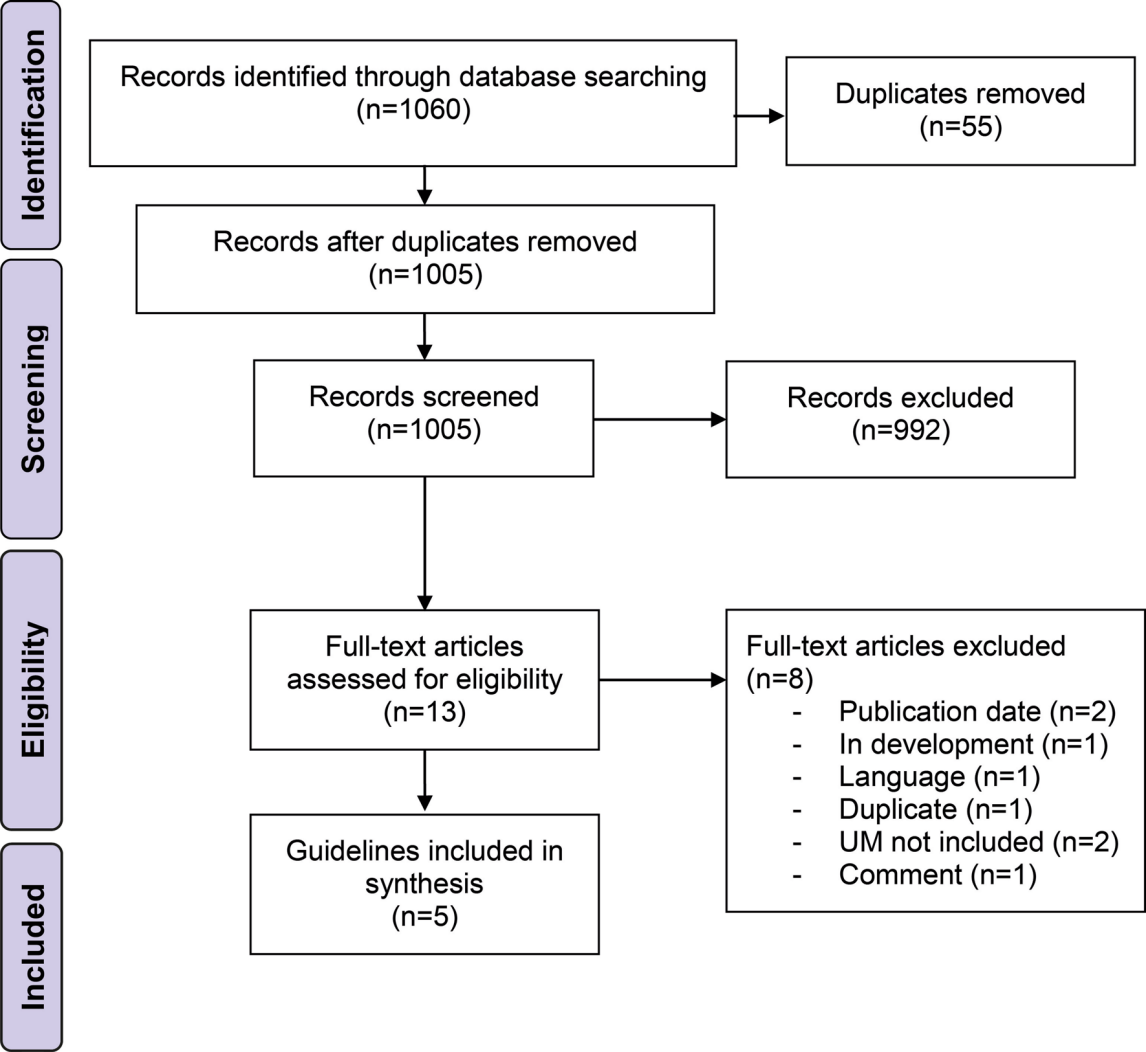
Flow chart of the guideline identification process according to the PRISMA guidelines

Our search in the databases and additional references revealed 1060 records (Fig. 1). After title and abstract screening and removal of duplicates, 13 records underwent full-text review. Two records were excluded since they were published before 2014 (Nag et al. 2003; Skalicky et al. 2008) and one was only available in French language (Mathis et al. 2018). Besides, one guideline was in development at the time of our search (National Institute for Health and Care Excellence 2019) and one more duplicate was identified (https://www.ncbi.nlm.nih.gov/books/NBK66047/). Another record was a comment on a guideline and was, therefore, excluded (Barker and Salama 2018). Furthermore, two guidelines were predominantly developed for the management of cutaneous melanoma and either did not address UM or did not cover it extensively or specifically enough to allow for an adequate appraisal with the chosen instruments (Leitlinienprogramm Onkologie 2019; Scottish Intercollegiate Guidelines Network 2017). Hence, five relevant guidelines were included in this comparison (National Cancer Institute 2019; Nathan et al. 2015; National Comprehensive Cancer Network 2018; Simpson et al. 2014; Weis et al. 2016). The publication date of the guidelines ranged from 2014 to 2018. Guidelines were available from consortia of the United States of America (*n* = 3), Canada (*n* = 1), and United Kingdom (UK) (*n* = 1) (Table 1). The majority of guidelines covered various aspects on UM, whereas one guideline exclusively focused on plaque brachytherapy of UM (Simpson et al. 2014). All included guidelines used distinct approaches to grade the level of evidence and to express the strength of their recommendations. The guideline assessment took place from June 2019 to August 2019. We determined a Fleiss’ Kappa of 0.088 (95% CI 0.069–0.108), indicating a slight overall interrater agreement concerning the assessment by AGREE II and AGREE-REX (Landis and Koch 1977).

**Table 1:** Overview of the identified UM guidelines

Title	Scope and key questions	National society and/or authors	Year	Methodological approach
Uveal Melanoma UK National Guidelines	The use and effectiveness of new technologies such as cytogenetics/genetic analysis for prognostication The appropriate pathway for the surveillance of patients following treatment for primary uveal melanoma The use and effectiveness of new technologies in the treatment of hepatic recurrence The use of systemic treatments	UK Melanoma Study Group, Uveal Melanoma Guideline Development Group Nathan et al. (2015)	2015	Evidence- and consensus-based
The American Brachytherapy Society consensus guidelines for plaque brachytherapy of uveal melanoma and retinoblastoma	To present the American Brachytherapy Society guidelines for plaque brachytherapy of choroidal melanoma and retinoblastoma	American Brachytherapy Society—Ophthalmic Oncology Task Force (Simpson et al. 2014)	2014	Evidence- and survey-based, consensus-based
Management of uveal melanoma: a consensus-based provincial clinical practice guideline	How should patients with uveal melanoma be staged at baseline? How should uveal melanoma be managed? What follow-up testing is required for uveal melanoma patients?	Weis et al. (2016)	2016	Evidence- and consensus-based
NCCN Clinical Practice Guidelines in Oncology: Uveal Melanoma	Explain differences in recommendations for molecular testing in uveal versus cutaneous melanoma Identify the most commonly used primary treatments for uveal melanoma and name the patient- and case-specific factors that would influence selection among these treatment options Describe the recommended imaging follow-up for patients with prior localized uveal melanoma, and explain why it differs from that for cutaneous melanoma	National Comprehensive Cancer Network (NCCN) Panel for Melanoma (Oncology 2018)	2018	Evidence- and consensus-based
Intraocular (Uveal) Melanoma Treatment	Treatment of intraocular melanoma. It is intended as a resource to inform and assist clinicians who care for cancer patients	National Cancer Institute (2019)	2019	Evidence-based, peer-reviewed

### AGREE II

#### Scope and purpose

**Fig. 2:**
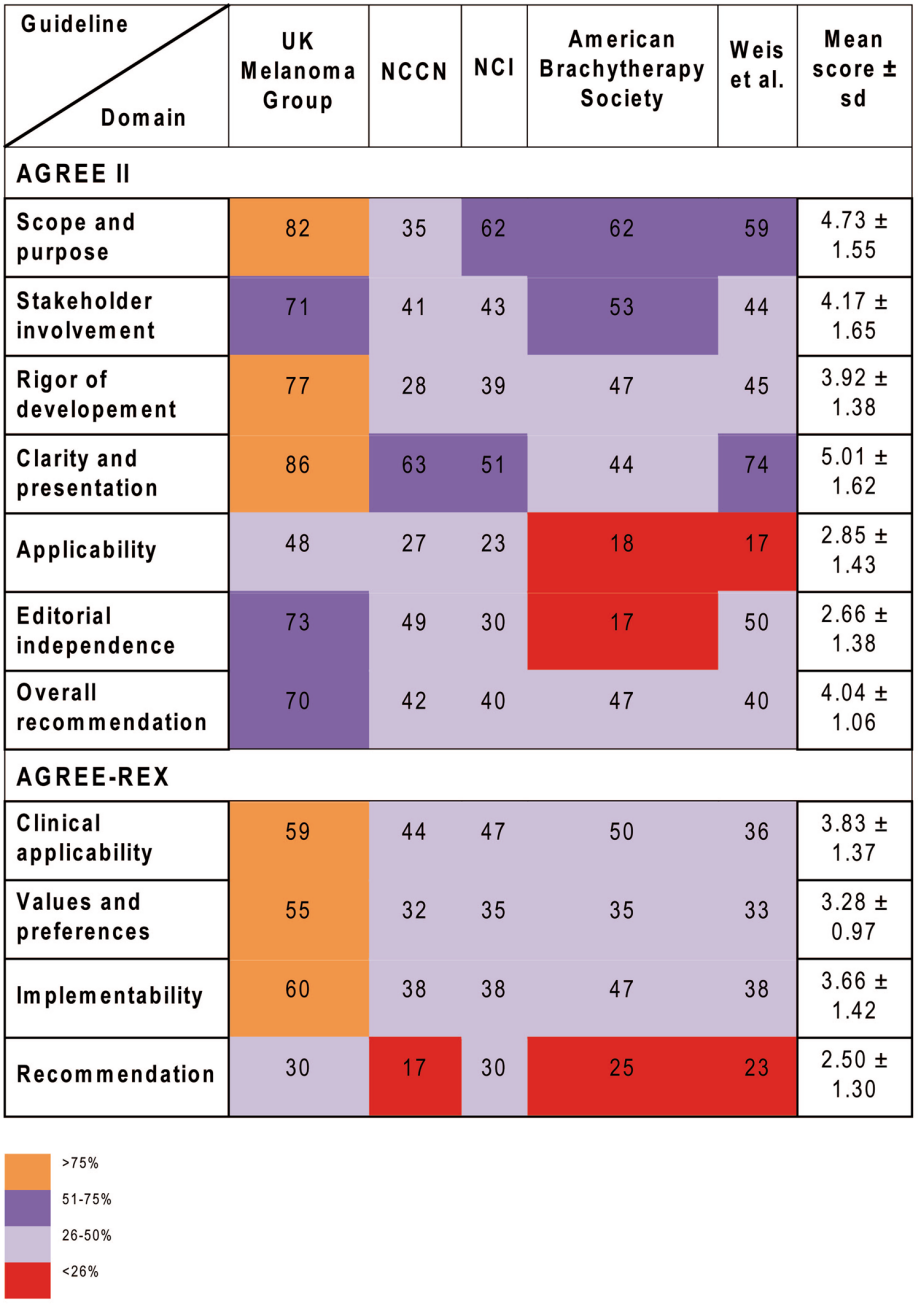
Heat-map showing an overview of the final AGREE II and AGREE-REX scores on UM guidelines as agreed upon by five independent evaluators

**Fig. 3:**
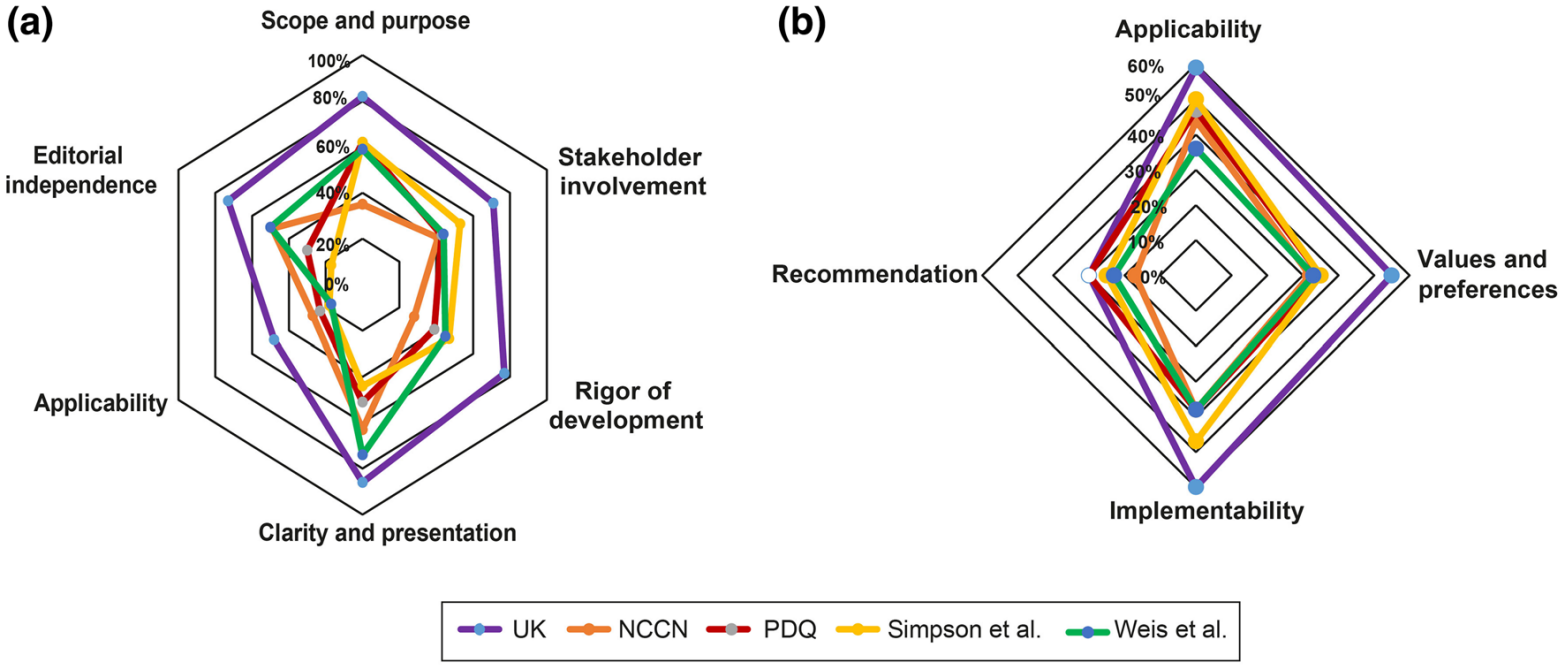
Network comparing the five different guidelines regarding the AGREE II (**a**) and AGREE REX (**b**) domains

This domain assesses whether the main objectives and questions of the guidelines and whether the population to whom the guideline is meant to apply were specifically described. It achieved an average score of 4.73 (± 1.55) (Supplementary Table S3). The guideline from the UK scored highest and fulfilled 82% of criteria whereas the guideline from the National Comprehensive Cancer Network (NCCN) from the US achieved only 35% of criteria (Fig. 2, 3a).

**Table 2:**
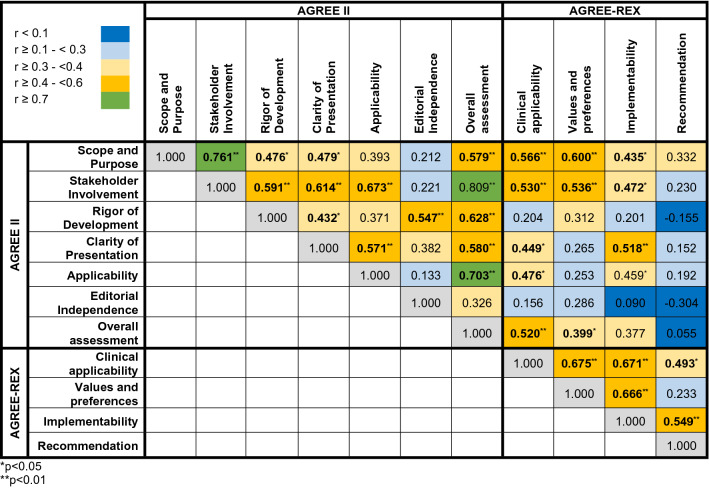
Correlations among the AGREE II and AGREE-REX domains

#### Stakeholder involvement

This domain evaluates whether the guideline was developed by appropriate stakeholders and represents the views of its intended users. Furthermore, it covers whether the target users of the guideline were clearly defined. The mean score of this domain was 4.17 (± 1.65) and the fulfilled values ranged from 71% for the UK guideline to intermediate values for the remaining guidelines (range 41–53%) (Fig. 2).

#### Rigor of development

This domain is about the methodological approaches of the guidelines and evaluates whether the identification of the evidence for the guideline was performed using systematic and transparent methods. Besides, this domain also assessed whether there was an explicit link between the recommendations and the supporting evidence, whether the guidelines had been externally reviewed and if a procedure for updating the guideline was available. It achieved an average rating of 3.92 (± 1.38). Besides, the UK guideline was evaluated by the reviewers to be of best methodological quality (77% of items fulfilled) in contrast to the remaining guidelines varying in terms of items fulfilled from 28 to 45%.

#### Clarity and presentation

This domain included the presentation and format of guidelines, i.e. whether the recommendations were specific and ambiguous, if key recommendations were easily identifiable and whether different options for the management of the condition were clearly presented. In general, all guidelines achieved high levels of fulfillment (5.01 ± 1.62) with the UK guideline being evaluated best with 86% of fulfilled criteria.

#### Application

This domain covers the processes related to guideline implementation, for instance facilitators, barriers, additional material provided and whether monitoring and/or auditing criteria were presented. This domain showed a mean score of 2.85 (± 1.43). The UK guideline achieved the highest score in comparison to the remaining guidelines (48%). The guidelines by Weis et al. and Simpson et al. achieved lowest possible scores with 17% and 18%, respectively.

#### Editorial independence

This domain focuses on funders and competing interests of experts involved in guideline development, i.e. whether competing interests of the development group were recorded and addressed. This domain achieved the lowest score with a mean of 2.66 (± 1.38). However, the UK guideline still achieved 73% of fulfilled criteria. The five identified guidelines significantly differed from each other (*p* = 0.038). No further subgroup differences were identified.

#### Overall assessment

This assessment rates the overall quality of the guidelines and whether the guideline would be recommended for use in practice. Overall, the guidelines achieved a mean score with 4.04 (± 1.06). The individual fulfilled criteria ranged from 40% (NCI guideline) to 70% (UK guideline). Hence, according to the assessment, only the UK guideline would be recommended whereas the remaining 4 guidelines are recommended with modifications only.

### AGREE-REX

#### Clinical applicability

This domain evaluates whether the guideline is evidence-based (i.e. based on a thorough review and assessing potential bias) as well as the degree to which the recommendations are applicable to the guideline’s target users’ practice context and patients. The guideline from the UK Melanoma group achieved the highest percentage value with 59%, and the guideline by Weis et al. the lowest with 36% (Fig. 3b, Supplementary Table S4). The mean score in this domain was 3.83 ± 1.37 (Fig. 2).

#### Values and preferences

This domain comprises four different items and refers to the relative importance that target users, patients, policy/decision-makers as well as guideline developers place on the outcomes of interest. Their values and preferences are important in guideline development as they influence whether recommendations are acceptable and adopted into practice. Therefore, this domain assesses if their views and its impact had been explored and considered in the formulation of the recommendations. The mean score was 3.28 ± 0.97 for this domain. All guidelines achieved a value ranging from 32 to 35%, except for the UK guideline which achieved 55% in this domain.

#### Implementability

The implementability domain includes the items “purpose” and “local application and adoption”. This domain assesses the suitability of the guideline recommendations for patients/populations, and/or the health care systems in which they are being implemented and if the degree of change from current practice was addressed. Furthermore, the guideline should articulate relevant factors important to its successful dissemination. Besides, the purpose item evaluates weather guideline recommendations aligned with the implementation goals of the guidelines. Again, the UK guideline achieved highest results with 60%, followed by the guideline of the American Brachytherapy Society (47%). The mean score was 3.66 ± 1.42.

#### Recommendations

This domain assessed whether the raters would recommend this guideline either in the appropriate context as well as in the rater’s context. Overall, none of the guidelines achieved sufficient values as they ranged from 17 to 30% and as the mean score was 2.50 ± 1.30.

### Correlations of the AGREE II and AGREE-REX domains

The majority of AGREE II domains significantly correlated with each other (Table 2). The domain “overall assessment” was significantly positively correlated with all other domains. “Stakeholder involvement” was highly positively correlated with the domain “overall assessment” (*r* = 0.81), the domain “scope and purpose” (*r* = 0.76), “clarity of presentation” (*r* = 0.48) and “rigor of development” (*r* = 0.48). Besides this, “clarity of presentation” was positively associated with “rigor of development” (*r* = 0.43) and “applicability” (*r* = 0.57). The AGREE-REX domains were all positively and statistically significantly correlated with each other, except for the domain “values and preferences” and the additional recommendation item.

## Discussion

UM is an orphan cancer condition of high unmet clinical need. Although UM differs from cutaneous melanoma both clinically and biologically, treatment options for advanced stages have largely been adopted from cutaneous melanoma, yet with much lower response rates and at the cost of high treatment-related toxicity (Heppt et al. 2017a,^2019^). In addition, numerous high-quality guidelines are available for the care of cutaneous melanoma, which facilitate the clinical and diagnostic algorithms in a standardized fashion (Pflugfelder et al. 2013; Swetter et al. 2019). In contrast, few studies have been published for UM and large randomized controlled trials are widely lacking. This makes it difficult to demonstrate the clinical effectiveness of interventions and to create a solid framework for evidence-based treatment decisions. A further barrier comes from the fact that the care of patients with UM occurs in a highly interdisciplinary setting, involving ophthalmologists, medical oncologists, interventional radiologists and dermato-oncologists. Patients suffering from rare cancers may have limited access to specialized cancer centers, potentially resulting in sub-optimal management and outcomes. These considerations highlight the value of high-quality guidelines in rare cancers even in the absence of high-quality evidence and underlines that special strategies need to be employed to synthesize evidence that is compatible with rigorous quality standards of guidelines (Pai et al. 2019).

In this study, we identified 5 guidelines for UM in a systematic literature search published within the previous 5 years and evaluated their methodological quality. Surprisingly, all of them were evidence-based and none was developed based on an expert consensus only. This contrasts with the fact that the domain “rigor of development” achieved rather low values (25–50%) in 4 out of 5 guidelines. This domain considers *inter alia* if systematic methods were applied to identify the evidence and if criteria for the literature search were clearly described and transparent. Thus, the majority of guidelines fell short of complying with these criteria. Similarly, the assessors gave low to intermediate ratings (25–50%) to the domains “overall recommendation”, “clinical applicability”, “values and preference”, and “implementability”, warranting improvement and special attention in future guideline efforts. The lowest values (≤ 25%) were observed for “applicability”, “editorial independence”, and “recommendation”. Applicability refers to how facilitators and barriers of the guideline application were discussed and if tools for monitoring or auditing the recommendations were provided. These parameters were not sufficiently addressed in any of the guidelines, possibly explaining the low ratings of this domain. The domain editorial independence aims at ensuring that the guideline is editorially independent from the funding body and that all conflicts of interest of guideline development members are correctly disclosed. The ratings for this domain were conspicuously low among the evaluated guidelines. On the one hand, it is possible that the editorial independency was present, but simply not indicated correctly. On the other hand, we cannot rule out the possibility that funding parties may have exerted an influence on the content of the guidelines or that the members of the guidelines group may have had conflicts of interest. Developing methodologically sound guidelines is costly and requires major financial, organizational and human resources. Especially in the case of orphan diseases such as UM, public and independent funding may not be available due to a low public awareness. Finally, consistently low values were achieved for “recommendation”, i.e. the usability of the recommendations made for a specific health-care context. Apparently, there was a major mismatch between the recommendations of the 5 guidelines with the specific care context of the assessor. Thus, the medical needs both of treating physicians and patients must be explored and aligned with the guideline recommendations, as has been performed for other orphan conditions (Boffin et al. 2018). Importantly, our evaluation did not evaluate the recommended therapies in the respective guideline but instead focused on their methodological quality.

Altogether, this analysis demonstrates that guidelines for UM as prime example of an orphan cancer have room for methodological improvement. The published UK guideline showed the best ratings in this study, suggesting that it may serve as good adaption basis for future guideline projects. In particular, the domains “applicability”, “editorial independence”, and “recommendation” should be improved to ensure that the guidelines give independent and context-specific guidance for clinicians.

## Electronic supplementary material

Below is the link to the electronic supplementary material.

Supplementary file1 (DOCX 20 kb)
