# Supplementary material for: Guidelines for uveal melanoma: a critical appraisal of systematically identified guidelines using the AGREE II and AGREE-REX instrument
Source: J Cancer Res Clin Oncol. 2020 Feb 8;146(4):1079–88. doi: 10.1007/s00432-020-03141-w (PMC7085474; doi:10.1007/s00432-020-03141-w)
Supplement: Supplementary file 1 — Supplementary file1 (DOCX 20 kb) [file 432_2020_3141_MOESM1_ESM.docx]

**Supplementary Table S1: Overview of the guideline databases searched for “uveal melanoma”, “ocular melanoma”, “Aderhautmelanom”, “iris melanoma”, “ciliary body melanoma”, and “choroidal melanoma”.**

| **Guideline database** | **Initial hits** |
| --- | --- |
| Arbeitsgemeinschaft der Wissenschaftlichen Medizinischen Fachgesellschaften: AWMF (https://www.awmf.org/leitlinien/leitlinien-suche.html) | 1 |
| Ärztliches Zentrum für Qualität in der Medizin: ÄZQ (leitlinien.de) | 0 |
| Arzneimittelkommission der deutschen Ärzteschaft (AkdÄ) <http://www.akdae.de> | 0 |
| National Institute for Health and Care Excellence: NICE (guidance.nice.org.uk/CG/Published) | 1 |
| Guidelines International Network: GIN (g-i-n.net) | 0 |
| Agency for Healthcare Research and Quality: NGC (guidelines.gov/) | 277 |
| Scottish Intercollegiate Guidelines Network: SIGN (sign.ac.uk/) | 0 |
| Dutch Guidelines (oncoline.nl/index.php?language=en) | 0 |
| Cross references | 2 |

**Supplementary Table S2: Overview of the search strategy in Medline and Embase via Ovid.**

| Search query in Medline (Ovid MEDLINE(R) and Epub Ahead of Print, In-Process & Other Non-Indexed Citations, Daily and Versions(R) 1946 to May 24, 2019; n=152 hits) |
| --- |
| 1. practice guideline.mp. or exp Practice Guideline/  2. health planning guideline.mp. or exp Health Planning Guidelines/  3. exp Guideline/ or guideline.mp. or exp Guideline Adherence/  4. guidance.mp.  5. evidence-based medicine.mp. or exp Evidence-Based Medicine/  6. exp Critical Pathways/ or care pathway.mp.  7. consensus development.mp. or exp Consensus/  8. 1 or 2 or 3 or 4 or 5 or 6 or 7  9. exp Eye Neoplasms/ or exp Uveal Neoplasms/ or exp Choroid Neoplasms/ or uveal melanoma.mp.  10. exp Uvea/ or uvea.mp.  11. exp Eye/  12. exp Iris/  13. ocular.mp.  14. exp Choroid/  15. 10 or 11 or 12 or 13 or 14  16. exp Melanoma/ or melanoma.mp.  17. 15 and 16  18. 9 or 17  19. 8 and 18 |
| Search query in Embase (Embase 1974 to 2019 May 24; n=627 hits) |
| 1. practice guideline.mp. or exp practice guideline/  2. health planning guidelines.mp. or exp health care planning/  3. guideline.mp. or exp practice guideline/  4. care pathway.mp. or exp clinical pathway/  5. guidance.mp.  6. consensus development.mp. or exp consensus development/ or exp decision making/ or exp consensus/  7. evidence-based medicine.mp. or exp evidence based medicine/  8. 1 or 2 or 3 or 4 or 5 or 6 or 7  9. uveal melanoma.mp. or exp uvea melanoma/  10. eye neoplasm.mp. or exp eye tumor/  11. choroid neoplasm.mp. or exp choroid tumor/  12. ocular neoplasm.mp.  13. exp eye/  14. exp iris/  15. ocular.mp.  16. exp choroid/  17. 13 or 14 or 15 or 16  18. exp melanoma/  19. exp uvea/  20. uvea tumor.mp. or exp uvea tumor/  21. 9 or 10 or 11 or 12 or 20  22. 13 or 14 or 15 or 16 or 19  23. 18 and 22  24. 21 or 23  25. 8 and 24  26. limit 25 to yr="2014 - 2019 |

**Supplementary Table S3: Overview of AGREE II mean scores (± standard deviation) of the 5 UM guidelines assessed by 5 independent reviewers on a scale ranging from 1 (lowest quality) to 7 (highest quality).**

| **Domain/item** | **UK Melanoma Group** | **NCCN** | **NCI** | **American Brachytherapy Society** | **Weis et al.** |
| --- | --- | --- | --- | --- | --- |
| **Scope and purpose** | | | | | |
| 1 Description overall objectives | 6.40 ± 0.89 | 2.60 ± 0.55 | 6.00 ± 1.23 | 5.00 ± 1.41 | 4.40 ± 1.82 |
| 2 Description health questions | 5.80 ± 2.17 | 4.20 ± 2.59 | 4.00 ± 2.35 | 4.80 ± 1.79 | 5.60 ± 1.34 |
| 3 Description of patient population at focus | 5.60 ± 1.67 | 4.40 ± 1.14 | 4.20 ± 2.28 | 4.40 ± 2.07 | 3.60 ± 1.95 |
| Overall percentage of scope and purpose | 82% | 35% | 62% | 62% | 59% |
| **Stakeholder involvement** | | | | | |
| 4 Relevant groups included in guideline group | 4.20 ± 1.92 | 5.40 ± 2.51 | 2.80 ± 2.49 | 5.60 ± 1.30 | 3.60 ± 1.67 |
| 5 Patient preferences considered | 5.60 ± 2.19 | 3.80 ± 1.92 | 2.60 ± 2.51 | 3.20 ± 1.30 | 2.40 ± 2.07 |
| 6 Target users deﬁned | 6.00 ± 1.73 | 3.20 ± 2.28 | 5.40 ± 1.14 | 3.80 ± 1.48 | 5.00 ± 1.87 |
| Overall percentage of stakeholder involvement | 71% | 41% | 43% | 53% | 44% |
| **Rigor of development** | | | | | |
| 7 Systematic literature research | 6.00 ± 1.73 | 3.00 ± 1.87 | 1.60 ± 1.34 | 5.20 ± 0.84 | 5.60 ± 1.67 |
| 8 Description of literature selection criteria | 5.00 ± 2.00 | 2.40 ± 0.89 | 2.80 ± 2.49 | 2.40 ± 1.52 | 5.20 ± 1.48 |
| 9 Strengths and limitations of evidence | 6.20 ± 1.10 | 2.80 ± 1.38 | 2.40 ± 1.67 | 2.20 ± 0.84 | 3.20 ± 1.30 |
| 10 Description methods for formulating recommendations | 6.20 ± 0.84 | 3.60 ± 3.13 | 2.00 ± 1.41 | 3.40 ± 1.95 | 2.60 ± 1.95 |
| 11 Side-effect, risks considered in recommendations | 5.60 ± 1.14 | 3.80 ± 1.48 | 3.40 ± 2.30 | 4.60 ± 0.89 | 3.20 ± 1.30 |
| 12 Link between evidence and recommendations | 5.60 ± 2.19 | 3.40 ± 2.07 | 4.60 ± 1.67 | 5.20 ± 1.10 | 4.40 ± 1.52 |
| 13 External review prior to publication | 6.40 ± 0.89 | 3.00 ± 1.87 | 3.80 ± 2.39 | 5.80 ± 1.30 | 4.00 ± 1.87 |
| 14 Procedure for updating provided | 3.80 ± 3.03 | 3.60 ± 3.13 | 6.00 ± 1.23 | 1.80 ± 1.10 | 1.20 ± 0.45 |
| Overall percentage of methodology | 77% | 28% | 39% | 47% | 45% |
| **Clarity and presentation** | | | | | |
| 15 Recommendation speciﬁc and unanimous | 6.00 ± 1.00 | 5.80 ± 2.17 | 4.20 ± 1.79 | 4.00 ± 0.70 | 5.60 ± 1.34 |
| 16 Clear presentation of different treatment options | 6.00 ± 1.00 | 6.00 ± 1.73 | 4.80 ± 1.92 | 4.20 ± 0.84 | 4.80 ± 1.30 |
| 17 Key recommendations easily identiﬁable | 6.40 ± 0.89 | 5.40 ± 2.51 | 3.20 ± 2.39 | 2.80 ± 1.30 | 6.00 ± 1.00 |
| 18 Tools for application available | 4.60 ± 1.82 | 2.20 ± 0.84 | 3.80 ± 2.95 | 2.00 ± 1.00 | 1.80 ± 1.30 |
| Overall percentage of clarity and presentation | 86% | 63% | 51% | 44% | 74% |
| **Applicability** | | | | | |
| 19 Discussion of organizational facilitators and barriers | 3.20 ± 0.84 | 4.20 ± 1.79 | 3.40 ± 2.41 | 1.80 ± 0.84 | 1.80 ± 0.84 |
| 20 Consideration of costs of guideline implementation | 3.20 ± 1.80 | 2.40 ± 0.89 | 1.80 ± 1.10 | 2.20 ± 0.84 | 1.80 ± 0.84 |
| 21 Inclusion of monitoring/auditing criteria | 4.60 ± 2.88 | 3.80 ± 2.28 | 3.40 ± 3.29 | 2.40 ± 2.61 | 2.60 ±2.51 |
| Overall percentage of applicability | 48% | 27% | 23% | 18% | 17% |
| **Editorial independence** | | | | | |
| 22 Editorial independence from funding body | 4.40 ± 1.82 | 3.60 ± 1.51 | 2.80 ± 2.68 | 2.60 ± 1.14 | 3.60 ± 2.19 |
| 23 Reporting of conﬂicts of interests | 6.40 ±0.89 | 5.80 ± 1.64 | 1.20 ± 0.45 | 1.40 ± 0.55 | 4.40 ± 2.41 |
| Overall percentage of editorial independence | 73% | 49% | 30% | 17% | 50% |
| **Total score** | 5.20 ± 1.30 | 4.20 ± 1.30 | 3.60 ± 0.55 | 3.80 ± 0.45 | 3.40 ± 0.55 |
| **Overall assessment** | 70% | 42% | 40% | 47% | 40% |

**Supplementary Table S4: Overview of AGREE-REX mean scores (± standard deviation) of the 5 UM guidelines assessed by 5 independent reviewers on a scale ranging from 1 (lowest quality) to 7 (highest quality).**

| **Item** | **UK Melanoma Group** | **NCCN** | **NCI** | **American Brachytherapy Society** | **Weis et al.** | **Overall assessment** |
| --- | --- | --- | --- | --- | --- | --- |
| **Clinical applicability** | | | | | | |
| Evidence | 4.20 ± 1.30 | 2.80 ± 1.30 | 4.20 ± 0.45 | 3.40 ± 2.07 | 2.00 ± 1.73 | 3.32 ± 1.60 |
| To users | 5.00 ± 1.41 | 5.00 ± 1.23 | 3.60 ± 1.95 | 4.60 ± 1.52 | 3.80 ± 1.30 | 4.40 ± 1.50 |
| To patients and populations | 4.40 ± 1.82 | 3.20 ± 1.48 | 3.60 ± 1.95 | 4.00 ± 1.87 | 3.60 ± 1.34 | 3.76 ± 1.62 |
| Overall percentage | 59% | 44% | 47% | 50% | 36% |  |
| **Values and preferences** | | | | | | |
| Of target users | 4.80 ± 1.30 | 4.00 ± 0.70 | 3.80 ± 0.84 | 4.00 ± 1.23 | 4.00 ± 0 | 4.12 ± 0.93 |
| Of patients and populations | 4.40 ± 1.82 | 3.40 ± 1.82 | 2.40 ± 0.89 | 3.20 ± 1.30 | 3.00 ± 1.00 | 3.28 ± 1.46 |
| Of policy and decision-makers | 3.20 ± 1.10 | 1.60 ± 0.55 | 2.80 ± 2.49 | 2.20 ± 1.10 | 2.00 ± 1.00 | 2.36 ± 1.41 |
| Of guideline developer | 4.80 ± 1.48 | 2.60 ± 0.89 | 3.40 ± 1.34 | 3.00 ± 1.23 | 3.00 ± 1.00 | 3.36 ± 1.35 |
| Overall percentage | 55% | 32% | 35% | 35% | 33% |  |
| **Implementability** | | | | | | |
| Purpose | 5.40 ± 1.34 | 4.60 ± 1.34 | 3.80 ± 1.92 | 4.60 ± 1.67 | 4.20 ± 1.64 | 4.52 ± 1.56 |
| Local application and adoption | 3.80 ± 1.92 | 2.00 ± 1.23 | 2.80 ± 2.34 | 3.00 ± 1.41 | 2.40 ± 1.14 | 2.80 ± 1.66 |
| Overall percentage | 60% | 38% | 38% | 47% | 38% |  |
| **Recommendation** | | | | | | |
| For use in appropriate context | 2.80 ± 2.39 | 2.00 ± 1.23 | 2.80 ± 0.84 | 2.20 ± 1.10 | 2.40 ± 0.89 | 2.44 ± 1.33 |
| For use in my context | 2.80 ± 2.39 | 2.00 ± 1.23 | 2.80 ± 0.84 | 2.80 ± 0.84 | 2.40 ± 0.89 | 2.56 ± 1.29 |
| Overall percentage | 30% | 17% | 30% | 25% | 23% |  |
